# Detection of COVID-19 from Chest X-ray Images Using Deep Convolutional Neural Networks

**DOI:** 10.3390/s21175940

**Published:** 2021-09-03

**Authors:** Natheer Khasawneh, Mohammad Fraiwan, Luay Fraiwan, Basheer Khassawneh, Ali Ibnian

**Affiliations:** 1 Department of Software Engineering, Jordan University of Science and Technology, P.O. Box 3030, Irbid 22110, Jordan; 2 Department of Computer Engineering, Jordan University of Science and Technology, P.O. Box 3030, Irbid 22110, Jordan; mafraiwan@just.edu.jo; 3 Department of Biomedical Engineering, Jordan University of Science and Technology, P.O. Box 3030, Irbid 22110, Jordan; fraiwan@just.edu.jo; 4 Department of Internal Medicine, Jordan University of Science and Technology, P.O. Box 3030, Irbid 22110, Jordan; basheerk@just.edu.jo (B.K.); amibnian@hotmail.com (A.I.)

**Keywords:** COVID-19, chest X-ray, deep learning, convolutional neural networks, diagnosis

## Abstract

The COVID-19 global pandemic has wreaked havoc on every aspect of our lives. More specifically, healthcare systems were greatly stretched to their limits and beyond. Advances in artificial intelligence have enabled the implementation of sophisticated applications that can meet clinical accuracy requirements. In this study, customized and pre-trained deep learning models based on convolutional neural networks were used to detect pneumonia caused by COVID-19 respiratory complications. Chest X-ray images from 368 confirmed COVID-19 patients were collected locally. In addition, data from three publicly available datasets were used. The performance was evaluated in four ways. First, the public dataset was used for training and testing. Second, data from the local and public sources were combined and used to train and test the models. Third, the public dataset was used to train the model and the local data were used for testing only. This approach adds greater credibility to the detection models and tests their ability to generalize to new data without overfitting the model to specific samples. Fourth, the combined data were used for training and the local dataset was used for testing. The results show a high detection accuracy of 98.7% with the combined dataset, and most models handled new data with an insignificant drop in accuracy.

## 1. Introduction

Coronavirus disease 2019 (COVID-19), which is caused by the SARS-CoV-2 virus, has wreaked havoc on humanity, especially healthcare systems. For example, recently, the wave of infections in India has caused a great number of families to seek care at home due to a lack of intensive care units. Worldwide, millions have succumbed to this pandemic and many more have suffered long- and short-term health problems. The most common symptoms of this viral syndrome are fever, dry cough, fatigue, aches and pains, loss of taste/smell, and breathing problems [1]. Other less common symptoms are also possible (e.g., diarrhea, conjunctivitis) [2]. Infections are officially confirmed using real-time reverse transcription polymerase chain reaction (RT-PCR) [3]. However, chest radiographs using plain chest X-rays (CXRs) and computerized tomography (CT) play an important role confirming the infection and evaluating the extent of damage incurred to the lungs. CXR and CT scans are considered major evidence for clinical diagnosis of COVID-19 [4].

Chest X-ray images are one of the most common clinical diagnosis methods. However, reaching the correct judgement requires specialist knowledge and experience. The strain on medical staff worldwide incurred by the COVID-19 pandemic, in addition to the already inadequate number of radiologists per person worldwide [5], necessitates innovative accessible solutions. Advances in artificial intelligence have enabled the implementation of sophisticated applications that can meet clinical accuracy requirements and handle large volumes of data. Incorporating computer-aided diagnosis tools into the medical hierarchy has the potential to reduce errors, improve workload conditions, increase reliability, and replace by enhance the workflow and reduce diagnostic errors by providing radiologists with references for diagnostics.

The fight against COVID-19 has taken several forms and fronts. Computerized solutions offer contactless alternatives to many aspects of dealing with the pandemic [6]. Some examples include robotic solutions for physical sampling, vital sign monitoring, and disinfection. Moreover, image recognition and AI are being actively used to identify confirmed cases not adhering to quarantine protocols. In this work, we propose an automatic diagnosis artificial intelligence (AI) system that is able to identify COVID-19-related pneumonia from chest X-ray images with high accuracy. One customized convolutional neural networks model and two pre-trained models (i.e., MobileNets [7] and VGG16 [8]) were incorporated. Moreover, CXR images of confirmed COVID-19 subjects were collected from a large local hospital and inspected by board-accredited specialists over a period of 6 months. These images were used to enrich the limited number of existing public datasets and form a larger training/testing group of images in comparison to the related literature. Importantly, the reported results come from testing the models with this completely foreign set of images in addition to evaluating the models using the fused aggregate set. This approach exposed any overfitting of the model to a specific set of CXR images, especially as some datasets contain multiple images per subject.

## 2. Background and Related Work

**Figure 1:**
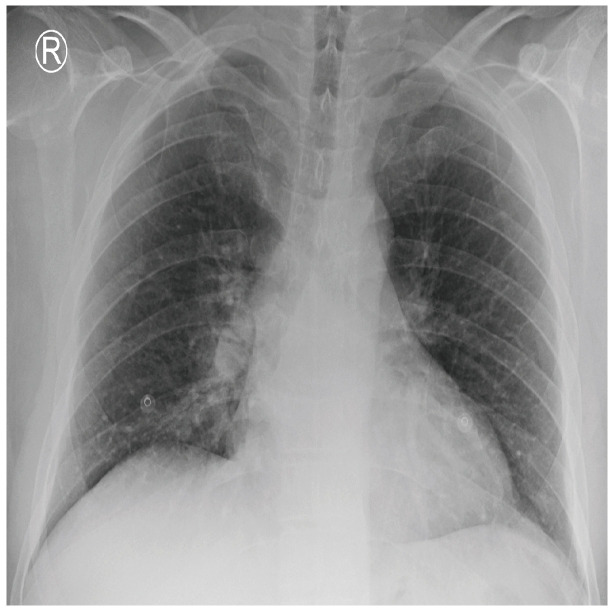
CXR of COVID-19 subject showing mild lung tissue involvement.

**Figure 2:**
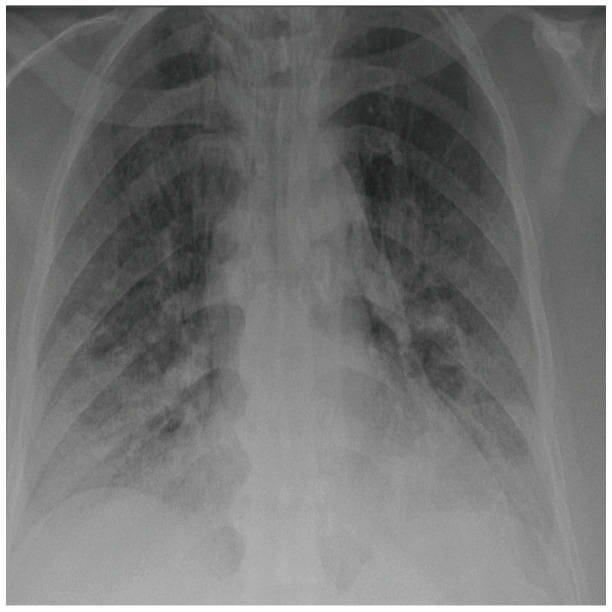
CXR of COVID-19 subject showing moderate to severe lung tissue involvement.

**Figure 3:**
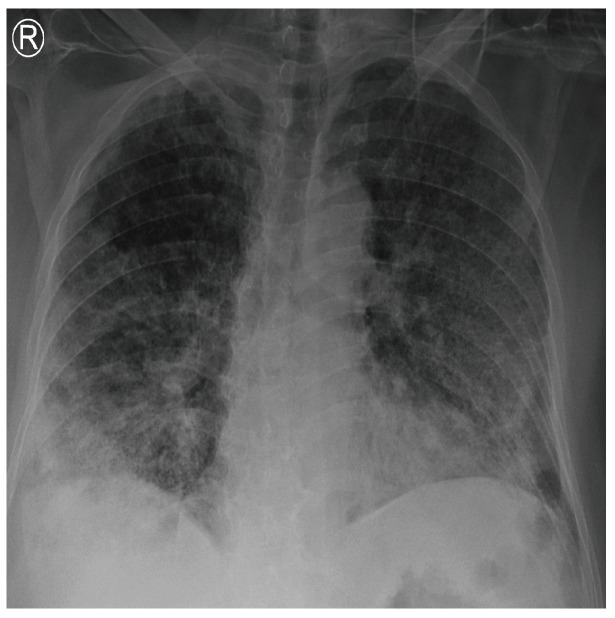
CXR of COVID-19 subject showing severe lung tissue involvement.

**Figure 4:**
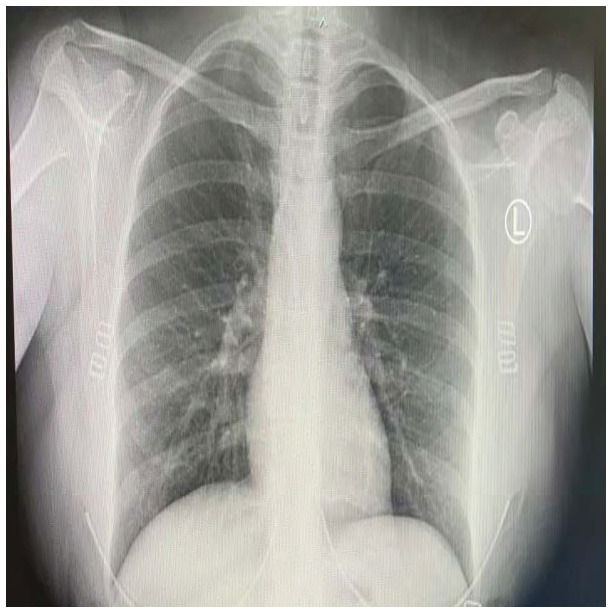
Normal CXR.

COVID-19 patients who have clinical symptoms are more likely to show abnormal CXR [9]. The main findings of recent studies suggest that these lung images display patchy or diffuse reticular–nodular opacities and consolidation, with basal, peripheral, and bilateral predominance [10]. For example, Figure 1 shows the CXR of a mild case of lung tissue involvement with right infrahilar reticular–nodular opacity. Moreover, Figure 2 shows the CXR of a moderate to severe case of lung tissue involvement. This CXR shows right lower zone lung consolidation and diffuse bilateral airspace reticular–nodular opacities, which are more prominent on peripheral parts of lower zones. Similarly, Figure 3 shows the CXR of a severe case of lung tissue involvement. This is caused by diffuse bilateral airspace reticular–nodular opacities that are more prominent on peripheral parts of the lower zones, and ground glass opacity in both lungs predominant in mid-zones and lower zones. On the other hand, Figure 4 shows an unremarkable CXR with clear lungs and acute costophrenic angles (i.e., normal).

AI, with its machine learning (ML) foundation, has taken great strides toward deployment in many fields. For example, Vetology AI [11] is a paid service that provides AI-based radiograph reports. Similarly, the widespread research and usage of AI in medicine have been observed for many years now [12,13]. AI-based web or mobile applications for automated diagnosis can greatly aid clinicians in reducing errors, provide remote and cheap diagnosis in poor undermanned underequipped areas, and improve the speed and quality of healthcare [14]. In the context of COVID-19 radiographs, ML methods are feasible to evaluate CXR images to detect the aforementioned markers of COVID-19 infection and the adverse effects on the state of the patients’ lungs. This is of special importance considering the fact that health services were stretched to their limits and sometimes to the brink of collapse by the pandemic.

Deep learning AI enables the development of end-to-end models that learn and discover classification patterns and features using multiple processing layers, rendering it unnecessary to explicitly extract features. The sudden spread of the COVID-19 pandemic has necessitated the development of innovative ways to cope with the rising healthcare demands of this outbreak. To this end, many recent models have been proposed for COVID-19 detection. These methods rely mainly on CXR and CT images as input to the diagnosis model [15,16]. Hemdan et al. [17] proposed the COVIDX-Net deep learning framework to classify CXR images as either positive or negative COVID-19 cases. Although they employed seven deep convolutional neural network models, the best results were 89% and 91% F1-scores for normal and positive COVID-19, respectively. However, their results were based on 50 CXR images only, which is a very small dataset to build a reliable deep learning system.

Several existing out-of-the-box deep learning convolutional neural network algorithms are available in the literature [18], and they have been widely used in the COVID-19 identification literature with and without modifications [15]. They provide track-proven image detection and identification capabilities in many disciplines and research problems. Some of the most commonly used models are: (1) GoogleNet, VGG-16, VGG-19, AlexNet, and LetNet, which are spatial exploitation-based CNNs. (2) MobileNet, ResNet, Inception-V3, and Inception-V4, which are depth based CNNs. (3) Other models include DenseNet, Xception, SqueezeNet, etc. These architectures can be used pre-trained with deep transfer learning (e.g., Sethy et al. [19]), or customized (e.g., CoroNet [20]).

Rajaraman et al. [21] used iteratively pruned deep learning ensembles to classify CXRs into normal, COVID-19, or bacterial pneumonia with a 99.01% accuracy. Several models were tested and the best results were combined using various ensemble strategies to improve the classification accuracy. However, such methods are mainly suitable for small numbers of COVID-19 images as the computational overhead of multiple model calculations is high, and there is no guarantee that they will retain their accuracy with large datasets [15,22]. Other works for three-class classification using deep learning were also proposed in this context. The studies by Ucar et al. [23], Rahimzadeh and Attar [24], Narin et al. [25], and Khobahi et al. [26] classify cases as COVID-19, normal, or pneumonia. Others replace pneumonia with a generic non-COVID-19 category [27,28], or severe acute respiratory syndrome (SARS) [29]. Less frequently, studies distinguish between viral and bacterial pneumonia in a four-class classification [18]. A significant number of studies conducted binary classification into COVID-19 or non-COVID-19 classes [19,30]. Although these methods achieved high accuracies (i.e., greater than 89%), the number of COVID-19 images from the total dataset is small. For example, Ucar et al. [23] used 45 COVID-19 images only. Moreover, subsequent testing of the models used a subset of the same dataset, which may give falsely improved results, especially as same subject may have multiple CXR images in the dataset.

## 3. Material and Methods

### 3.1. Subjects

The selected images were acquired from locally recorded chest X-rays of COVID-19 patients in addition to a publicly available dataset [31]. The combination of two datasets adds greater credibility to the developed identification models. This is because training/validation was performed on one set, and the testing was performed on a different dataset. In addition, it increased the size of the dataset, which is a problem with most of the related literature.

The first group of images was obtained locally at King Abdullah University Hospital, Jordan University of Science and Technology, Irbid, Jordan. The study was approved by the institutional review board (IRB 91/136/2020) at King Abdullah University Hospital (KAUH). Written informed consent was sought and obtained from all participants (or their parents in case of underage subjects) prior to any clinical examinations. The dataset included 368 subjects (215 male, 153 female) with a mean ± SD age of 63.15 ± 14.8. The minimum subject age was 31 months and maximum age was 96 years. All subjects had at least one positive RT-PCR test and were in need of hospital admittance as determined by the specialists at KAUH. The hospital stay ranged from 5 days to 6 weeks with some subjects passing away (exact number not available). The CXR images were taken after at least 3 days of hospital stay to ensure the existence of lung abnormalities, which were confirmed by the participating specialists. The CXR images were reviewed using the MicroDicom viewer version 3.8.1 (see https://www.microdicom.com/, accessed on: 28 May 2021), and exported as high-resolution images (i.e., 1850 × 1300 pixels).

The second group of images is publicly available [31], and was produced by the fusion of three separate datasets: (1) COVID-19 chest X-ray dataset [32]. (2) The Radiological Society of North America (RSNA) dataset [33]. (3) The U.S. National Library of Medicine (USNLM) Montgomery County X-ray set [34]. At the time of performing the experiments, the dataset contained 2295 CXR images (1583 normal and 712 COVID-19), which were used in this work. However, the dataset is continuously being updated [35].

### 3.2. Deep Learning Models

Deep learning is the current trend and most prolific AI technique used for classification problems [15]. It has been used widely and successfully in a range of applications, especially in the medical field. The next few paragraphs describe the models used in this work.

1. 2D sequential CN CNN models are one class in the deep learning literature. They are a special class of feedforward neural networks that have been found to be very useful in analyzing multidimensional data (e.g., images) [18]. However, CNNs conserve memory relative to multilayer perceptrons by sharing parameters and using sparse connections. The input images are transformed into a matrix to be processed by the various CNN elements. The model consists of several alternating layers of convolution and pooling (see Table 1), as follows:
  Convolutional layer
    The convolutional layer determines the features of the various patterns in the input. It consists of a set of dot products (i.e., convolutions) applied to the input matrix. This step creates an image processing kernel containing a number of filters, which outputs a feature map (i.e., motifs). The input is divided into small windows called receptive fields, which are convolved with the kernel using a specific set of weights. In this work, a 2D convolution layer was used (i.e., using the CONV2D class).
  Pooling layer
    This down-sampling layer reduces the spatial dimensions of the output volume by reducing the number of feature maps and network parameters. Moreover, pooling helps in improving the generalization of the model by reducing overfitting [36]. The output from this step is a combination of features invariant to translational shifts and distortions [37].
  Dropout
    Overfitting is a common problem in neural networks. Hence, dropout is used as a strategy to introduce regularization within the network, which eventually improves generalization. It works by randomly ignoring some hidden and visible units. This has the effect of training the network to handle multiple independent internal representations.
  Fully connected layer
    This layer accepts the feature map as input and outputs nonlinear transformed output via an activation function. This is a global operation that works on features from all stages to produce a nonlinear set of classification features. The rectified linear unit (ReLU) was used in this step as it helps in overcoming the vanishing gradient problem [38].
2. Pre-trained models
  MobileNets
    The MobileNets model [7] is a resource-limited CNN architecture, which was chosen in this work with an eye on future mobile applications for disease diagnosis. It uses depth-wise separable convolutions, which significantly reduces the number of parameters. MobileNets was open-sourced by Google to enable the development of low-power, small, and low-latency applications for mobile environments.
  VGG-16
    VGG-16 [8] is a representative of the many models existing in the literature. It has gone through various refinements to improve its accuracy performance and resources consumption (e.g., VGG-19). The VGG model is a spatial exploitation CNN with 19 layers, 3 × 3 filters (computationally efficient), 1 × 1 convolution in between the convolution layers (for regularization), and max-pooling after the convolution layer. The model is known for its simplicity [18].

### 3.3. Model Implementation

The models were implemented and evaluated using the Keras [39] high-level application program interface (API) of TensorFlow 2 [40]. The experiments were run on a Dell Precision 5820 Tower (Dell Inc., Round Rock, TX, USA) with Intel Xeon W-2155, 64GB of RAM (Intel Inc., Santa Clara, CA, USA), and 16GB Nvidia Quadro RTX5000 GPU (Nvidia Inc., Santa Clara, CA, USA).

## 4. Results and Discussion

Four different approaches were used to evaluate the three deep learning models. First, only the public dataset was used to train and test the models. Second, the fused dataset was used to test and train the models (i.e., the sets were combined together and treated as one without any distinction). Third, the public dataset was used for training the model and the locally collected dataset was used for testing. This approach shows the ability of the model to generalize to new data and avoid overfitting to specific images/subjects. Fourth, the combined (i.e., fused) dataset was used for training and local dataset for testing. Table 2 shows the number of training and testing subjects used for each approach. Note that the local dataset did not include normal CXR images as those are abundantly available. The confusion matrices resulting from testing were analyzed to produce several standard performance measures. These include accuracy, specificity, sensitivity, F1-score, and precision as defined in Equations (1)–(5).

(1) $$Accuracy=\frac{t_{p}+t_{n}}{t_{p}+f_{n}+f_{p}+t_{n}}$$

(2) $$Sensitivity=\frac{t_{p}}{t_{p}+f_{n}}$$

(3) $$Specificity=\frac{t_{n}}{t_{n}+f_{p}}$$

(4) $$F1score=\frac{2\times t_{p}}{2\times t_{p}+f_{p}+f_{n}}$$

(5) $$Precision=\frac{t_{p}}{t_{p}+f_{p}}$$

where $t_{p}$: true positive, represents the subjects correctly classified in predefined (positive) class. $f_{n}$: false negative, represents the subjects misclassified in the other (negative) class. $f_{p}$: false positive, represents the subjects misclassified in predefined (positive) class. $t_{n}$: true negative, represents the subjects correctly classified in the other (negative) class.

**Figure 5:**
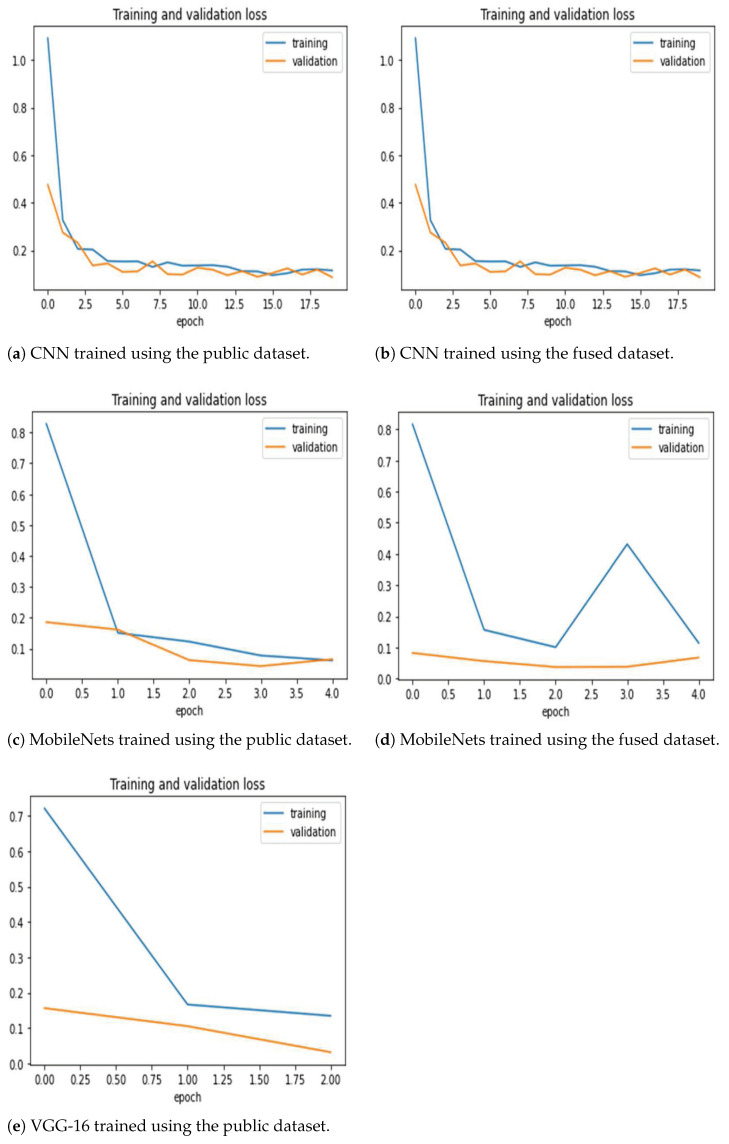
Training and validation loss for the three architectures trained using the public and the fused datasets. Note that VGG-16 trained on the fused dataset ended after one epoch only, hence there is no corresponding figure. The models are able to properly fit training data and improve with experience (as seen in validation curves).

**Figure 6:**
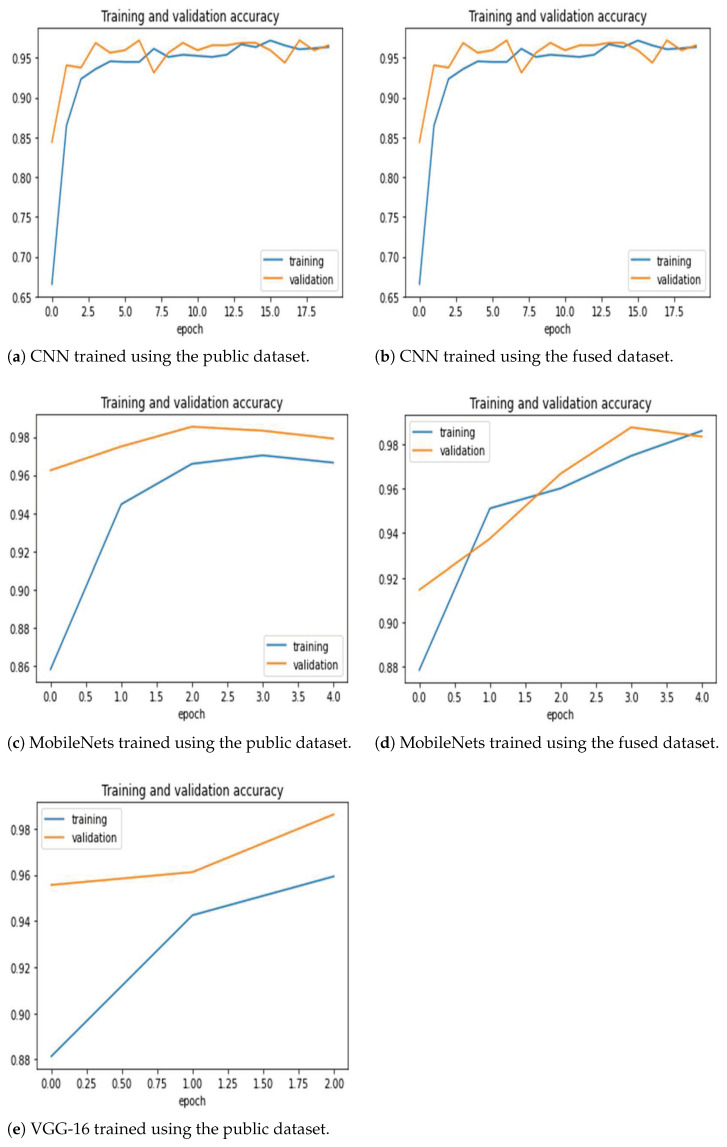
Training and validation accuracy for the three architectures trained using the public and the fused datasets. Note that VGG-16 trained on the fused dataset ended after one epoch only, hence there is no corresponding figure. The models are able to properly fit training data and improve with experience (as seen in validation curves).

Figure 5 shows the training and validation loss for the two model training methods. The 2D CNN model required more epochs to reach the appropriate accuracy improvement, but the training was smooth with little oscillation. Moreover, the other two models required very few epochs (e.g., VGG-16 required one epoch with the fused dataset, hence the missing plot). Figure 6 shows the training and validation accuracy. The figures generally show that the models are able to properly fit training data and improve with experience. It is clear that the MobileNets and VGG-16 models achieve superior and high classification accuracy.

The testing dataset (i.e., the locally collected COVID-19 CXR images) is different from the training dataset.

### 4.1. 2D Sequential CNN

Table 3 and Table 4 show the values for the performance evaluation metrics and the corresponding confusion matrices for the 2D sequential CNN model. The architecture achieved the best accuracy of 96.1% over all training and testing methods. However, the accuracy drops sharply to 79% when the testing was carried out using a database (i.e., the locally collected COVID-19 CXR images) different from the training one (i.e., the public dataset). This indicates the failure of the model to generalize to new data, and that there may be subtle or obscure differences between the images from the two datasets. This is further confirmed by the fact that normal images (see Table 4c), which were taken from the public dataset, were mostly correctly classified. The source of errors came from false negative classifications (i.e., type II errors). However, the accuracy improved to 89.3%, when a separate part of the testing dataset was included in the training. Still, most of the errors were type II (see Table 4d). This is a model performance mismatch problem of the custom CNN, which is typically caused by unrepresentative data samples. However, since the other models were trained on the same data, then this reason could be discounted. The MobileNets and VGG-16 models were employed using transfer learning, which inherently reduces overfitting. Moreover, these models are larger and deeper than the custom CNN, which due to overparameterization can lead to better generalization performance [41].

### 4.2. MobileNets

Table 5 and Table 6 show the values for the performance evaluation metrics and the corresponding confusion matrices for the MobileNets model. It achieved accuracy values between 97.1% and 98.7%, which shows stability when faced with new data, and the ability to generalize. Errors, although few, were caused by misclassifying COVID-19 CXR images as normal. However, the type I errors increased slightly (Table 6c).

### 4.3. VGG-16

Table 7 and Table 8 show the values for the performance evaluation metrics and the corresponding confusion matrices for the VGG-16 model. The model achieved the best accuracy over all models (i.e., 99%) when the fused dataset was used for training and the local dataset was used for testing, which indicates its ability to capture various properties from different sets. However, it fell behind MobileNets slightly when the training dataset (i.e., the public dataset) was different from the testing dataset. Moreover, the model achieved the highest accuracy (98.7%) with the fused dataset for both training and testing. However, MobileNets achieved slightly higher accuracy when trained and tested with the public dataset alone. Such slight performance differences when the dataset is augmented with data from other sources may need further investigation. The confusion matrices show that, for VGG-16, the majority of errors are type I over all evaluation methods, which is different from the CNN or MobileNets errors (i.e., type II). Improving VGG-16’s handling of normal images should cut the error rate significantly.

### 4.4. Comparison to Related Work

Table 9 shows a performance comparison of deep learning studies in binary classification using CXR images. Some studies did not report the accuracy as their datasets were largely imbalanced. Although most related studies reported high accuracy values, a common theme among them is the lack of a significant number of COVID-19 cases for this type of classification model. For example, Narin et al. [25] mention that the excess number of normal images resulted in higher accuracy in all of those models. This is useless considering the fact that very few differences exist among normal images of lungs across different subjects. Similarly, Hemdan et al. [17] stated the limited number of COVID-19 X-ray images as the main problem in their work. Moreover, the dataset that we included in this work contains only one image per subject, unlike other datasets which include more images than subjects. In addition, special consideration was paid to the type of cases included in the dataset, because the effect of COVID-19 on the lungs does not necessarily appear immediately with symptoms and it may take a few days.

The literature on deep learning for medical diagnosis in general and COVID-19 classification in particular is vast and expanding. However, large datasets are required to truly have reliable generalized models. We believe that development of mobile and easy access applications that capture and store data on the fly will enable better data collection and improved deep learning models.

## 5. Conclusions

Global disasters bring people together and spur innovations. The current pandemic and the worldwide negative consequences should present an opportunity to push forward technological solutions that facilitate everyday life. In this study, we have collected chest X-ray images from hospitalized COVID-19 patients. These data will enrich the current available public datasets and enable further refinements to the systems employing them. Moreover, deep learning artificial intelligence models were designed, trained, and tested using the locally collected dataset as well as public datasets, both separately and combined. The high accuracy results present an opportunity to develop mobile and easy access applications that improve the diagnosis accuracy, reduce the workload on strained health workers, and provide better healthcare access to undermanned/underequipped areas. Future work will focus on this avenue as well as development and evaluation of multiclass classification models.

## Figures and Tables

**Table 1:** Summary of the CNN models used in this work.

Layer	Output Shape	No. of Parameters
CONV2D-1	(None, 150, 150, 32)	2432
MaxPooling2D-1	(None, 75, 75, 32)	0
Dropout-1	(None, 75, 75, 32)	0
Conv2D-2	(None, 75, 75, 64)	51,264
MaxPooling2D-2	(None, 37, 37, 64)	0
Dropout-2	(None, 37, 37, 64)	0
Flatten	(None, 87,616)	0
Dense-1	(None, 256)	22,429,952
Dropout-3	(None, 256)	0
Dense-2	(None, 1)	257

**Table 2:** The number of training and testing subjects used for each of the evaluation approaches.

Approach	Training		Testing	
	COVID-19	Normal	COVID-19	Normal
Public dataset	545	1266	167	317
Fused dataset	842	1266	238	317
Public dataset for training and local dataset for testing	545	1266	368	317
Fused dataset for training and local dataset for testing	842	1266	368	317

**Table 3:** Performance evaluation metrics for the customized CNN model. Acc.: Accuracy, Sens.: Sensitivity, Spec.: Specificity, Prec.: Precision.

Dataset	Acc.	Sens.	Spec.	F1-Score	Prec.
Public dataset	96.1%	92.8%	97.8%	94.2%	95.7%
Fused dataset	93.7%	85.7%	99.7%	92.1%	99.5%
Public dataset for training and local dataset for testing	79%	62.8%	97.8%	76.2%	97.1%
Fused dataset for training and local dataset for testing	89.3%	80.4%	99.7%	89%	99.7%

**Table 4:** The confusion matrices resulting from the customized CNN model. Positive refers to confirmed COVID-19 case.

(a) Public Dataset			
		Predicted diagnosis	
		Positive	Negative
Actual	Positive	155	12
	Negative	7	310
**(b) Fused Public and Local Datsets**			
		Predicted diagnosis	
		Positive	Negative
Actual	Positive	204	34
	Negative	1	316
**(c) Public Dataset for Training and Local Dataset for Testing**			
		Predicted diagnosis	
		Positive	Negative
Actual	Positive	231	137
	Negative	7	310
**(d) Fused Dataset for Training and Local Dataset for Testing**			
		Predicted diagnosis	
		Positive	Negative
Actual	Positive	296	72
	Negative	1	316

**Table 5:** Performance evaluation metrics for the customized MobileNets model. Acc.: Accuracy, Sens.: Sensitivity, Spec.: Specificity, Prec.: Precision.

Dataset	Acc.	Sens.	Spec.	F1-Score	Prec.
Public dataset	98.3%	98.2%	98.4%	97.6%	97%
Fused dataset	97.1%	92.8%	99.4%	95.7%	98.7%
Public dataset for training and local dataset for testing	98%	97.6%	98.4%	98.1%	98.6%
Fused dataset for training and local dataset for testing	98.7%	98.1%	99.4%	98.8%	99.4%

**Table 6:** The confusion matrices resulting from the customized MobileNets model. Positive refers to confirmed COVID-19 case.

(a) Public Dataset			
		Predicted diagnosis	
		Positive	Negative
Actual	Positive	164	3
	Negative	5	312
**(b) Fused Public and Local Datsets**			
		Predicted diagnosis	
		Positive	Negative
Actual	Positive	155	12
	Negative	2	315
**(c) Public Dataset for Training and Local Dataset for Testing**			
		Predicted diagnosis	
		Positive	Negative
Actual	Positive	359	9
	Negative	5	312
**(d) Fused Dataset for Training and Local Dataset for Testing**			
		Predicted diagnosis	
		Positive	Negative
Actual	Positive	361	7
	Negative	2	315

**Table 7:** Performance evaluation metrics for the customized VGG-16 model. Acc.: Accuracy, Sens.: Sensitivity, Spec.: Specificity, Prec.: Precision.

Dataset	Acc.	Sens.	Spec.	F1-Score	Prec.
Public dataset	97.1%	98.2%	96.5%	95.9%	93.7%
Fused dataset	98.7%	99.2%	98.4%	98.5%	97.9%
Public dataset for training and local dataset for testing	97.2%	97.8%	96.5%	97.4%	97%
Fused dataset for training and local dataset for testing	99%	99.5%	98.4%	99.1%	98.7%

**Table 8:** The confusion matrices resulting from the customized VGG-16 model. Positive refers to confirmed COVID-19 case.

(a) Public Dataset			
		Predicted diagnosis	
		Positive	Negative
Actual	Positive	164	3
	Negative	11	306
**(b) Fused Public and Local Datsets**			
		Predicted diagnosis	
		Positive	Negative
Actual	Positive	236	2
	Negative	5	312
**(c) Public Dataset for Training and Local Dataset for Testing**			
		Predicted diagnosis	
		Positive	Negative
Actual	Positive	360	8
	Negative	11	306
**(d) Fused Dataset for Training and Local Dataset for Testing**			
		Predicted diagnosis	
		Positive	Negative
Actual	Positive	366	2
	Negative	5	312

**Table 9:** Performance comparison of deep learning studies in binary COVID-19 diagnosis (i.e., positive or negative) using CXR images. Some studies did not report the accuracy as their datasets were largely imbalanced. All websites were last accessed on 28 May 2021.

Study	No. of COVID-19 Images and Database	Method	Accuracy
Singh et al. [42]	50, https://github.com/ieee8023/covid-chestxray-dataset	MADE-based CNN	94.7%
Sahinbas et al. [43]	50, https://github.com/ieee8023/covid-chestxray-dataset	VGG16, VGG19, ResNet, DenseNet, InceptionV3	80%
Medhi et al. [44]	150, https://www.kaggle.com/bachrr/covid-chest-xray	Deep CNN	93%
Narin et al. [25]	341, https://github.com/ieee8023/covid-chestxray-dataset	InceptionV3, ResNet50, ResNet101	96.1%
Sethy et al. [19]	48, https://www.kaggle.com/andrewmvd/convid19-X-rays	most available models (e.g., DenseNet, ResNet)	95.3%
Minaee et al. [30]	71, https://github.com/ieee8023/covid-chestxray-dataset	ResNet18, ResNet50, SqueezeNet, DenseNet-121	–
Maguolo et al. [45]	144, https://github.com/ieee8023/covid-chestxray-dataset	AlexNet	–
Hemdan et al. [17]	25, https://github.com/ieee8023/covid-chestxray-dataset	VGG19, ResNet, DenseNet, Inception, Xception	90%
This work	712+368, doi.org/10.21227/x2r3-xk48+local	2D CNN, VGG16, MobileNets	up to 99%

## Data Availability

The dataset generated and/or analyzed during the current study is available from the corresponding author on reasonable request. The dataset will be made public in a separate data article.

## Abbreviations

The following abbreviations are used in this manuscript:

AI	artificial intelligence
COVID-19	coronavirus disease 2019
CT	computerized tomography
CXR	chest X-rays
KAUH	King Abdullah University Hospital
RT-PCR	real-time reverse transcription polymerase chain reaction
SARS	severe acute respiratory syndrome
